# Distinct clinical characteristics of adolescent idiopathic scoliosis with asymmetrical ESR1 expression in paraspinal muscle progenitor cells

**DOI:** 10.1002/jsp2.70018

**Published:** 2024-11-26

**Authors:** Hanlong Xin, Wenyuan Sui, Wenhua Mao, Junlin Yang, Xiexiang Shao

**Affiliations:** ^1^ Department of Orthopedic Surgery Taizhou Hospital of Zhejiang Province affiliated to Wenzhou Medical University Zhejiang China; ^2^ Spine Center Xin Hua Hospital Affiliated to Shanghai Jiao Tong University School of Medicine Shanghai China; ^3^ Department of Orthopedic Surgery XianJu People's Hospital, Zhejiang Southeast Campus of Zhejiang Provincial People's Hospital, Affiliated Xianju's Hospital, Hangzhou Medical College Zhejiang China

**Keywords:** adolescent idiopathic scoliosis, clinical characteristics, ESR1, muscle progenitor cells, paraspinal muscles

## Abstract

**Background:**

Previous studies found decreased ESR1 expression of concave paraspinal muscle progenitor cells could contribute to the initiation and progression of adolescent idiopathic scoliosis (AIS). The current study investigated the clinical characteristics of AIS with asymmetrical ESR1 expression in paraspinal muscle progenitor cells.

**Materials and Methods:**

Bilateral deep paraspinal muscle progenitor cells were obtained from 25 consecutive eligible female patients with AIS. RT‐qPCR was performed to evaluate the expression of ESR1. The demographic data (the age at surgery, height, weight, BMI, and age at initiation), posteroanterior and lateral radiographs data (Risser sign, Cobb angle, apical vertebral rotation, and location of apical vertebra), and MR imaging data (bilateral paraspinal muscle CSA ratio and bilateral fatty component ratio) were collected. The correlation between asymmetrical ESR1 expression of paraspinal muscle progenitor cells and the aforementioned clinical characteristics were analyzed.

**Results:**

Twelve out of twenty‐five patients (48%) showed bilateral ESR1 expression ratio (convex/concave) more than 1.5 folds, and they were divided into the ESR1 asymmetry group. When compared with the ESR1 symmetry group, patients in the ESR1 asymmetry group showed significantly more severe scoliosis (*p* = 0.041), more hypoplastic concave paraspinal muscle (*p* = 0.015), and more muscular fatty infiltration in the concave side (*p* = 0.034). The bilateral ESR1 expression ratio was significantly correlated with Cobb angle (*r* ^2^ = 0.282, *p* = 0.006), bilateral paraspinal muscle CSA ratio (*r* ^2^ = 0.253, *p* = 0.011), and bilateral fatty component ratio (*r* ^2^ = 0.248, *p* = 0.011).

**Conclusion:**

There were 48% of AIS patients with significantly decreased ESR1 expression in concave paraspinal muscle progenitor cells (convex/concave>1.5 folds), while patients with more asymmetrical ESR1 expression showed more hypoplastic paraspinal muscle and fatty infiltration on the concave side, and more severe scoliotic deformity.

## Graphical abstract

Clinical characteristics of AIS with asymmetrical ESR1 expression in paraspinal muscle progenitor cells were investigated. There were 48% AIS patients with significantly decreased ESR1 expression in concave paraspinal muscle progenitor cells, while asymmetrical ESR1 expression was correlated with asymmetrical paraspinal muscle CSA, asymmetrical fatty infiltration, and more severe scoliotic deformity.

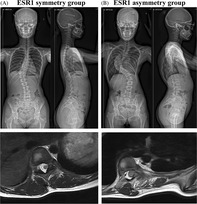

## 1 INTRODUCTION

Adolescent idiopathic scoliosis (AIS) is a complex three‐dimensional spinal deformity of unknown etiology that occurs during adolescence.^1^ It is characterized clinically by a coronal plane Cobb angle greater than 10° along with abnormal sagittal alignment and vertebral rotation.^1^ AIS has an incidence rate of 2%–3% among adolescents, with females being affected 1.5–3 times more frequently than males.^2^, ^3^ Approximately two‐thirds of AIS patients experienced continuous progression of spinal deformity during the rapid growth of adolescence.^3^ It can result in lower back pain, spinal degeneration, trunk imbalance, and even impair cardiopulmonary function.^3^, ^4^ Therefore, there is an urgent need for in‐depth research into the etiology of AIS to enable early intervention and prevent the initiation and progression of AIS.

Asymmetrical paraspinal muscles in AIS patients have been revealed by MRI studies,^5^, ^6^ biomechanical tests,^7^ and histopathological researches.^8^, ^9^, ^10^ Genetic factor that contributes to imbalanced paraspinal muscle could be a significant factor in the initiation and progression of AIS.^11^ Previous studies found that there was asymmetric expression of specific genes including PAX3,^12^ MYOD1,^13^ MT2,^14^ ADIPOQ,^15^ and H19^15^ of bilateral paraspinal muscles. Although these defective genes in paraspinal muscle could predispose patients with severe scoliotic deformity, the detailed mechanism of these genes for the initiation and progression of scoliosis remains to be explored. Recently, we found significantly decreased expression of ESR1 in muscle progenitor cells from concave paraspinal muscle.^16^ Decreased ESR1 impaired myogenic differentiation of muscle progenitor cells and contributed to imbalanced bilateral para‐spinal muscles, thus leading to spinal instability and curve progression of AIS.^16^ Thus, ESR1 was a potential risk gene with a mechanism proved. However, the relationship between ESR1 in muscle progenitor cells and the clinical characteristics of AIS patients remains unknown.

Thus, the current study was performed to investigate the clinical significance of asymmetrical ESR1 expression of paraspinal muscle progenitor cells. Especially, this study will focus on the relationship among asymmetrical ESR1 expression, demographic data, scoliotic deformity, asymmetrical paraspinal muscle and fatty infiltration in AIS patients. We hypothesized that asymmetrical ESR1 expression of paraspinal muscle progenitor cells was closely associated with clinical phenotypes of AIS patients.

## 2 MATERIALS AND METHODS

### 2.1 Subjects

Twenty‐five consecutive eligible subjects with AIS who underwent corrective operation from December 2019 to December 2021 were included. The detailed inclusion criteria were: (1) female participants aged 10 to 18 years; (2) diagnosed as AIS with Cobb angle more than 40 degrees; (3) underwent posterior fixation corrective surgical procedures. Those who previously underwent scoliosis correction or other spinal surgery were excluded. Approval for the study was obtained from the ethics committee of the local institution (Approval No. XHEC‐D‐2019‐093), and signed informed consent forms were obtained from all participants and their legal guardians. This study was in accordance with the Declaration of Helsinki. During surgery, bilateral deep paraspinal muscles were removed from the level of the apical vertebra at the major curve (the structural curve with the largest Cobb angle), following previously established protocols.^12^, ^13^, ^17^ This harvesting procedure posed no additional risk to the patients.

### 2.2 Isolation and identification of muscle progenitor cells

Isolation of muscle progenitor cells was performed as described previously.^16^, ^18^ In summary, muscle tissues were sliced into 1 mm^3^ pieces and then subjected to enzymatic digestion with collagenase II (Worthington Biochemical, 700–800 U/mL, cat#LS004177) for 1 h, followed by a subsequent digestion with a mixture of collagenase II and dispase (Life Technologies, 11 U/mL, cat#17105‐041) for 30 min. The digested mixture was then passed through a 20‐gauge needle 10 times and filtered through a 40‐μm cell strainer (BD Falcon, cat#352340). Erythrocytes were removed using red blood cell lysis (Thermo Fisher Scientific, cat#00‐433‐57). Then staining was performed with PE‐Cy5 anti‐human CD45 (BD Pharmingen, cat#555484, 1:25), Percp‐Cy5.5 anti‐human CD31 (BioLegend, cat#303132, 1:100), AF‐488 anti‐human CD29 (BioLegend, cat#303016, 1:100), and BV421 anti‐human CD56 (BD, cat#562751, 1:100) for 45 min at 4°C. Muscle progenitor cells were purified by gating CD29+/CD56+/CD45‐/CD31‐with Aria III or Influx (BD Biosciences).

Immunofluorescent staining of marker gene PAX7 was performed to confirm the purity of obtained cells. Total RNA extraction was carried out utilizing TRIzol Reagent (Invitrogen, cat#15596‐018) following the manufacturer's guidelines, followed by reverse transcription using MuLV reverse transcriptase (NEB, cat#M0253L) at 42°C for 60 min.

### 2.3 Quantitative PCR reactions

Quantitative PCR reactions were conducted using FastStart Universal SYBR Green Master (Roche, cat#4913914001) in the ABI Q6 real‐time PCR system (ABI), with GAPDH employed as the internal control. The primer sequences for RT‐qPCR are provided below: GAPDH‐F: 5′‐CAAGGCTGAGAACGGGAAGC‐3′; GAPDH‐R: 5′‐AGGGGGCAGAGATGATGACC‐3′; ESR1‐F: 5′‐CCCACTCAACAGCGTGTCTC‐3′; ESR1‐R: 5′‐CGTCGATTATCTGAATTTGGCCT‐3′.

### 2.4 Western blot assay

For Western blot, samples were firstly lysed by ice‐cold RIPA lysis for 10 min (Beyotime, cat#P0013C). The obtained proteins were then separated via SDS‐PAGE and transferred onto PVDF membranes. After being blocked with a 5% BSA solution for 1 h, the membranes were then incubated overnight at 4°C with primary antibodies targeting ESR1 (Abcam, cat#A19665) or GAPDH (CST, cat#2118L). Chemiluminescence was utilized to image the target proteins following incubation with the corresponding secondary antibodies for 1 h. Then obtained images were analyzed by Image J.

### 2.5 Demographic and imaging data evaluation

The age at surgery, height, weight, BMI, and age at initiation were recorded. According to standard preoperative standing posteroanterior and lateral radiographs, patients' Risser sign, Cobb angle, apical vertebral rotation, and location of apical vertebra in major curve were also collected.

A 1.5 T magnetic resonance imaging system (Siemens Healthineers; MAGNETOM Aera; Germany) was used for deep paraspinal muscle evaluation. Whole spine T2‐weighted axial images were obtained. The slice thickness was 4.0 mm with a 0.68 mm gap between each slice. The field of view for the scan was 400 * 400 mm. The slice showing apical vertebra in the major curve was identified for subsequent analysis by ImageJ ver.1.3 software. The bilateral cross‐sectional area (CSA) of deep paraspinal muscle including multifidus, semispinalis, and rotator muscles was outlined. The paraspinal muscle CSA ratio was defined as the ratio of paraspinal muscle CSA on the convex side to that on the concave side. The bilateral CSA of fat components (FC) in the total deep paraspinal muscle was also contoured. FC% was defined as FC CSA/(FC CSA + paraspinal CSA), which was a universal indicator to describe fatty infiltration.^19^ The ratio of FC% on the convex side to that on the concave side was also calculated for each patient. Two orthopedic spine surgeons who unaware of subject information independently measured the MR images twice. Intra‐observer variability was examined with one observer conducting measurements twice, while inter‐observer variability was assessed with a separate observer.

### 2.6 Statistical analysis

Statistical analysis was performed using SPSS version 19.0 for Windows (SPSS Inc., Chicago, IL, USA). The Shapiro–Wilk test was used to determine the normality of continuous data. Student's t test was used to compare data from the ESR1 symmetry and ESR1 asymmetry groups. The associations between ESR1 expression ratio and Cobb angle, as well as muscle CSA ratio and fatty infiltration, were evaluated using Pearson's correlation coefficient. Significance was attributed to results where the *p* value was below 0.05. Data were expressed as mean ± standard deviation.

## 3 RESULTS

Twenty‐five eligible female patients (mean age, 13.4 ± 1.3 years old) were enrolled in the study. The mean height was 160.4 ± 7.3 cm, with a mean weight of 46.8 ± 9.1 kg, and a mean BMI of 18.1 ± 3.0 kg/m^2^. The average age at initiation was 11.5 ± 2.0 years old. The mean Risser sign of the enrolled patients was grade 2.5 ± 1.3, and the mean Cobb angle of the major curve was 61.7 ± 18.4 degrees. The mean apical vertebral rotation according to Nash‐Moe classification was 1.6 ± 0.6. Apical vertebra in major curve were located in thoracic vertebra in 17 out of 25 patients, while 8 out of 25 patients had apical vertebra in major curve in lumbar vertebra. The patients' demographic data and clinical characteristics were tabulated in Table 1.

**TABLE 1:** Demographic and clinical characteristics of all subjects.

Parameters	Values
Age at surgery, years	13.4 ± 1.3
Height, cm	160.4 ± 7.3
Weight, kg	46.8 ± 9.1
BMI, kg/m^2^	18.1 ± 3.0
Age at initiation, years	11.5 ± 2.0
Risser sign	2.5 ± 1.3
Cobb angle, degrees	61.7 ± 18.4
Apical vertebral rotation	1.6 ± 0.6
Location of major curve	
Thoracic	17 (68)
Lumbar	8 (32)

*Note*: Quantitative data are described as mean ± standard deviation, whereas qualitative data are expressed as number (percentage).

**FIGURE 1:**
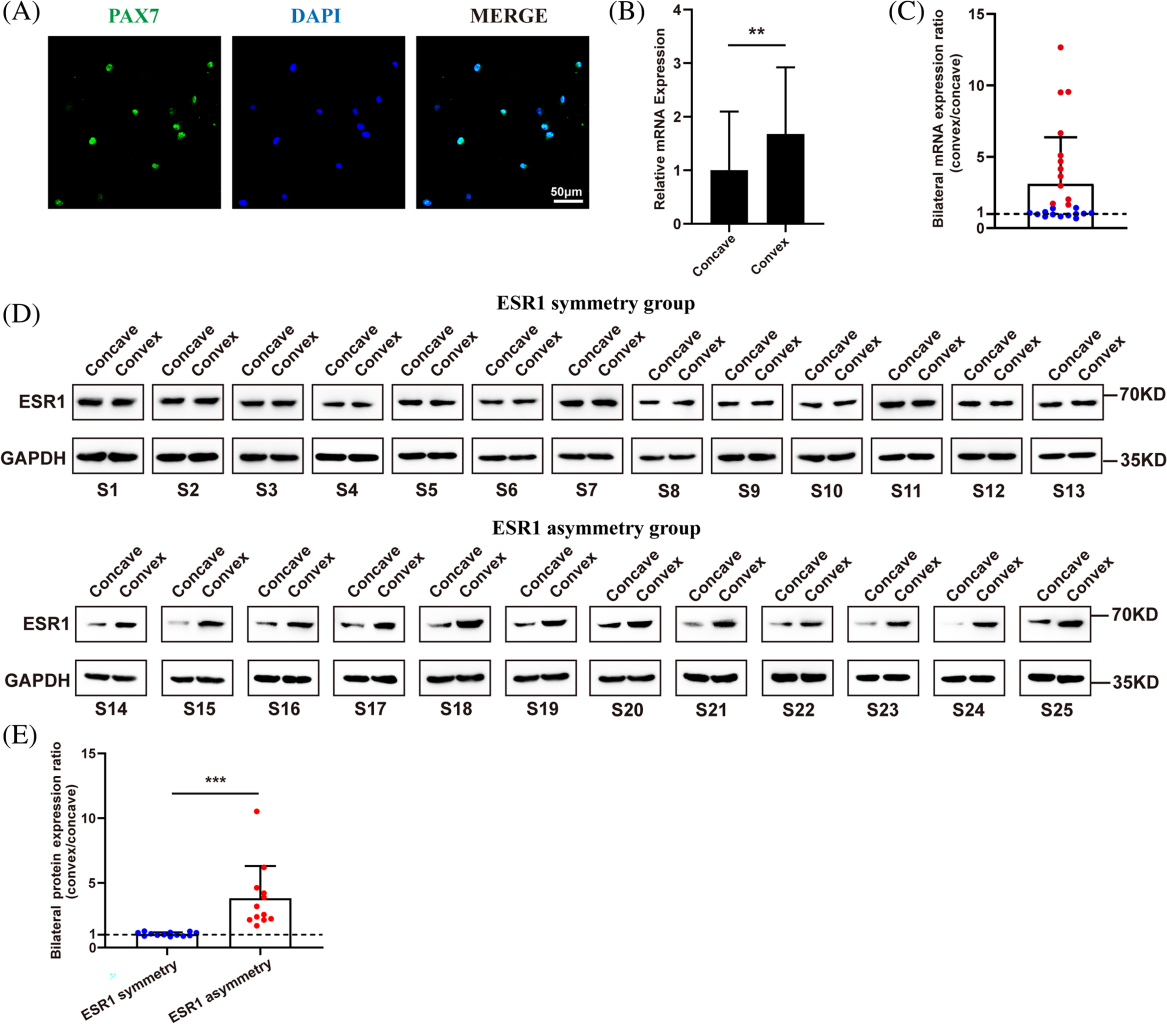
Comparison of ESR1 expression from bilateral paraspinal muscle progenitor cells. (A). Immunofluorescent staining of PAX7 for obtained cells. Scale bar, 50 μm; (B). Comparison of ESR1 mRNA expression from bilateral sides. ** indicated *p* < 0.01; (C). Scatter plot of bilateral mRNA expression ratio of ESR1 for each patient. Red points indicated ESR1 asymmetry group, while blue points indicated ESR1 symmetry group. (D). ESR1 protein level of samples from bilateral paraspinal muscle in ESR1 asymmetry group and ESR1 symmetry group. GAPDH was served as control. (E). Comparison of bilateral ESR1 protein expression ratio (convex/concave) in ESR1 asymmetry group and ESR1 symmetry group. Red points indicated ESR1 asymmetry group, while blue points indicated ESR1 symmetry group. *** indicated *p* < 0.001.

Immunofluorescent staining of PAX7 confirmed that the purified obtained cells were muscle progenitor cells (Figure 1A). The muscle progenitor cell from the concave side showed significantly decreased mRNA expression of ESR1 than the convex side (Figure 1B). When we compared bilateral mRNA expression ratio (convex/concave), we found that not all of the patients showed decreased ESR1 mRNA expression level of concave muscle progenitor cells (Figure 1C). Twelve out of twenty‐five patients (48%) showed a bilateral expression ratio (convex/concave) greater than 1.5 folds (Figure 1C), while the others showed the relative same bilateral expression level. Thus, we further divided these patients into two groups. Those who with a bilateral ESR1 mRNA expression ratio (convex/concave) greater than 1.5 folds were assigned to the ESR1 asymmetry group, while the others were assigned to the ESR1 symmetry group. The Western blot of those samples from bilateral paraspinal muscles also revealed a consistent result with RT‐qPCR of ESR1 expression level in the ESR1 symmetry and ESR1 asymmetry groups (Figure 1D,E). Then further analysis was performed to explore the differences between these two groups, including demographic data and imaging data.

**FIGURE 2:**
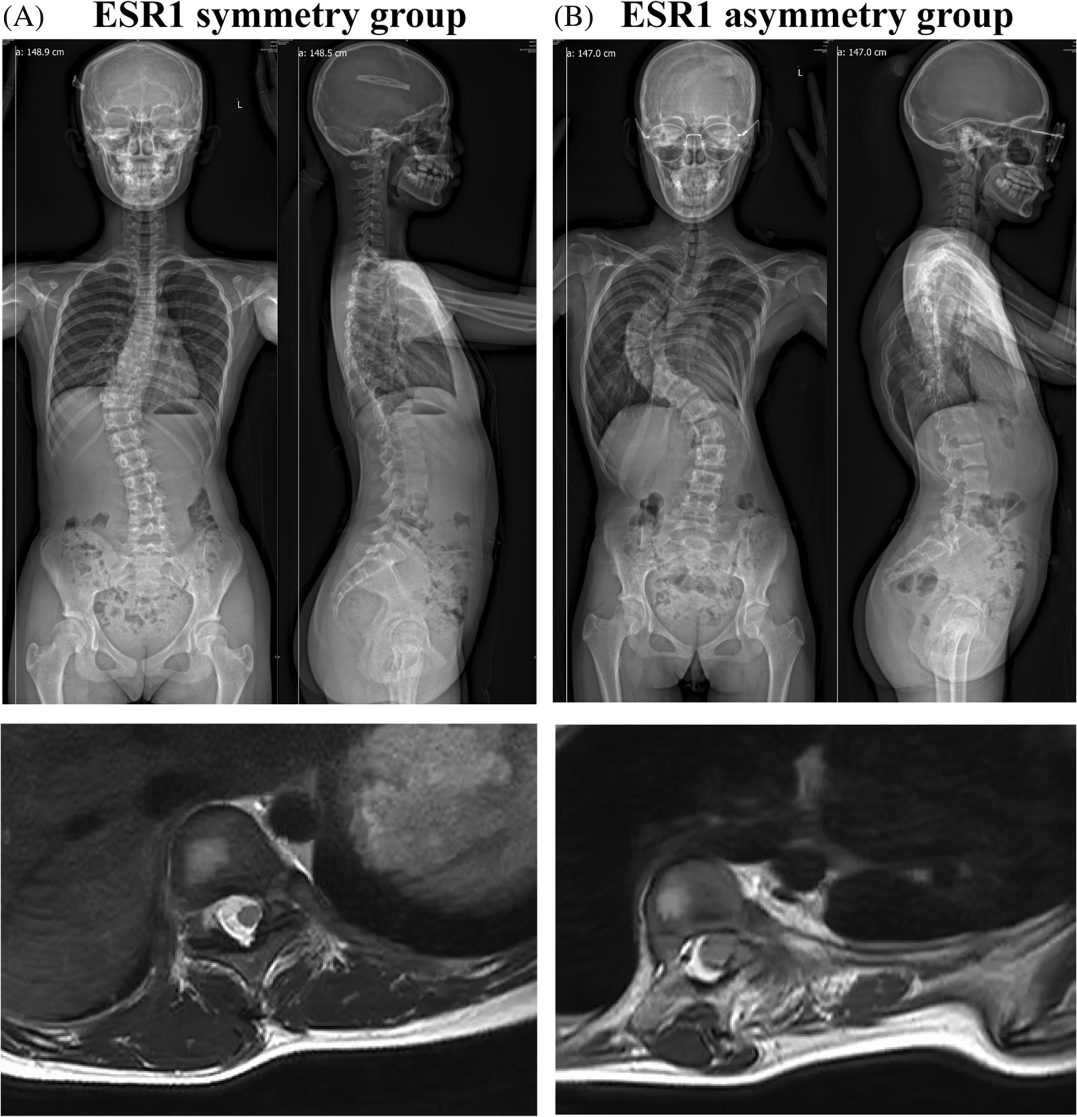
Representative standard preoperative standing posteroanterior/lateral radiographs and MR imaging showing apical vertebra in major curve in ESR1 symmetry group (A) or ESR1 asymmetry group (B).

There was no difference of age of surgery (13.2 ± 1.0 vs. 13.5 ± 1.4 years old, *p* = 0.612), height (159.5 ± 6.4 vs. 161.5 ± 8.0 cm, *p* = 0.513), weight (46.7 ± 7.7 vs. 46.9 ± 10.4 kg, *p* = 0.975), BMI (18.4 ± 3.1 vs. 17.8 ± 2.9 kg/m^2^, *p* = 0.523), age at initiation (11.5 ± 1.9 vs. 11.5 ± 2.0 years old, *p* = 0.963), Risser sign (2.6 ± 1.3 vs. 2.3 ± 1.3, *p* = 0.606), apical vertebral rotation (1.7 ± 0.7 vs. 1.5 ± 0.5, *p* = 0.480), location of apical vertebra in major curve (*p* = 0.471) between ESR1 symmetry and ESR1 asymmetry groups (Table 2). However, patients in the ESR1 asymmetry group showed significantly more severe scoliosis (Cobb angle, 69.6 ± 19.2 vs. 54.4 ± 14.2 degrees, *p* = 0.041), more decreased concave paraspinal muscle CSA (bilateral muscle CSA ratio, 1.4 ± 0.3 vs. 2.4 ± 1.8, *p* = 0.015), and more increased concave FC% (bilateral FC% ratio, 0.5 ± 0.1 vs. 0.6 ± 0.2, *p* = 0.034) than those in the ESR1 symmetry group (Table 2 and Figure 2).

**TABLE 2:** Comparison of patients with or without abnormal ESR1 expression.

	ESR1 symmetry group	ESR1 asymmetry group	*p* value
Age of surgery, years	13.2 ± 1.0	13.5 ± 1.4	0.612
Height, cm	159.5 ± 6.4	161.5 ± 8.0	0.513
Weight, kg	46.7 ± 7.7	46.9 ± 10.4	0.975
BMI, kg/m^2^	18.4 ± 3.1	17.8 ± 2.9	0.523
Age at initiation, years	11.5 ± 1.9	11.5 ± 2.0	0.963
Risser sign	2.6 ± 1.3	2.3 ± 1.3	0.606
Cobb angle, degrees	54.4 ± 14.2	69.6 ± 19.2	0.041
Apical vertebral rotation	1.7 ± 0.7	1.5 ± 0.5	0.480
Location of apical vertebra in major curve			
Thoracic	9 (75)	8 (62)	0.471
Lumbar	3 (25)	5 (38)	
Muscle CSA ratio (convex/concave)	2.4 ± 1.8	1.4 ± 0.3	0.015
FC% ratio (convex/concave)	0.6 ± 0.2	0.5 ± 0.1	0.034

*Note*: Quantitative data are described as mean ± standard deviation, whereas qualitative data are expressed as number (percentage).

Abbreviations: CSA, cross‐sectional area; FC, fat components.

**FIGURE 3:**
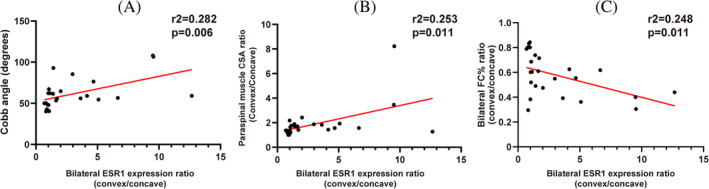
Correlation analysis of bilateral ESR1 expression ratio and related parameters. (A). Correlation analysis of bilateral ESR1 expression ratio and Cobb angle (*r* ^2^ = 0.282, *p* = 0.006); (B). Correlation analysis of bilateral ESR1 expression ratio and bilateral paraspinal muscle CSA ratio (*r* ^2^ = 0.253, *p* = 0.011); (C). Correlation analysis of bilateral ESR1 expression ratio and bilateral FC% ratio (*r* ^2^ = 0.248, *p* = 0.011).

To further explore the clinical significance of asymmetrical ESR1 expression of paraspinal muscle progenitor cells, correlation analysis between bilateral ESR1 expression ratio and related parameters were performed. There was a positive correlation between bilateral ESR1 expression ratio and Cobb angle (*r* ^2^ = 0.282, *p* = 0.006) (Figure 3A). Bilateral ESR1 expression ratio was also significantly correlated with bilateral paraspinal muscle CSA ratio (*r* ^2^ = 0.253, *p* = 0.011) (Figure 3B), and bilateral FC% ratio (*r* ^2^ = 0.248, *p* = 0.011) (Figure 3C). Thus, patients with more asymmetrical ESR1 expression showed more hypoplastic paraspinal muscle and fatty infiltration on the concave side, and more severe scoliotic deformity.

## 4 DISCUSSION

The current study evaluated the bilateral ratio of ESR1 expression of paraspinal muscle progenitor cells in AIS patients, and investigated the clinical characteristics of those with asymmetrical ESR1 expression of paraspinal muscle progenitor cells in AIS patients. We found that there were 48% of AIS patients with significantly decreased ESR1 expression of concave paraspinal muscle progenitor cells (convex/concave >1.5 folds), while patients with more asymmetrical ESR1 expression showed more hypoplastic paraspinal muscle and fatty infiltration on the concave side, and more severe scoliotic deformity.

Paraspinal muscle imbalance hypothesis is one of the important etiological theories for AIS.^16^ In previous investigations, paraspinal muscle imbalance identified by MRI scans,^5^, ^20^ biomechanical assessments,^7^ and muscle histology^21^ has been reported to be closely associated with AIS, while asymmetrical paraspinal muscles have been found to significantly correlate with the severity of scoliotic deformity.^7^ Thus, paraspinal muscles might play a pivotal role in the initiation and progression of AIS. The development of paraspinal muscle was facilitated by the myogenesis of muscle progenitor cells.^22^ Our previous study demonstrated that the reduced expression of ESR1 hindered the differentiation process of muscle progenitor cells on the concave side, thereby exacerbating the onset and improvement of AIS.^16^ However, the relationship between ESR1 in muscle progenitor cells and clinical features of AIS patients remains unknown. There is still a lack of substantial evidence to bridge imbalanced ESR1 expression to clinical phenotype to the support paraspinal muscle imbalance hypothesis for AIS.

One of the primary findings of the current study was that it unraveled the importance of asymmetrical ESR1 expression of bilateral paraspinal muscle progenitor cells for AIS from a clinical perspective. The patients with more asymmetrical bilateral ESR1 expression were accompanied by a larger Cobb angle, hypoplastic concave paraspinal muscle, and more muscular fatty infiltration on the concave side. As is known, muscle progenitor cells play a crucial role in rapid skeletal muscle growth during adolescent.^22^, ^23^ Muscle progenitor cell‐specific Esr1 KO mice showed significantly impaired muscle growth and development both in vivo and in vitro.^16^ Thus, the results of the current study provided additional evidence to support the imbalanced paraspinal muscle hypothesis for AIS as follows: (1) Impaired ESR1 expression in concave paraspinal muscle progenitor cells contributes to myogenesis defects, and thus causing relative hypoplastic paraspinal muscle and more muscular fatty infiltration in the concave side; (2) Then imbalanced bilateral para‐spinal muscles could apply asymmetric loading to the spine and lead to initial spinal instability/curvature^24^; (3) According to the Hueter‐Volkmann principle, the epiphyseal plates on the concave side have increased pressure that results in decreased growth, while less pressure on the convex side leads to accelerated vertebral growth^25^; (4) The asymmetric loading from para‐spinal muscle would cause asymmetrical vertebral growth and progressive wedging deformity, with the development of a scoliotic curve.^25^ Thus, the current study made the imbalanced paraspinal muscle hypothesis more convincing for the initiation and development of AIS.

Overall, the findings of this study shed light on the importance of targeting paraspinal muscle to prevent the initiation and development of AIS. For AIS patients with asymmetrical ESR1 expression of muscle progenitor cells, attention should be paid to applying a more aggressive treatment strategy to the concave paraspinal muscles. To our best knowledge, functional exercise and bracing are routinely performed as conservative treatments for AIS.^26^ However, the prolonged treatment course and time‐consuming nature of these treatments often result in poor medical compliance. Since decreased ESR1 expression on concave paraspinal muscle progenitor cells could contribute to the spinal deformity of AIS, pharmacological treatment to reactivate ESR1 signaling seems to be a simpler and more practical approach. Reactivation of ESR1 signaling in concave paraspinal muscle by selective estrogen receptor modulator Raloxifene has been introduced as a potential treatment strategy to alleviate the curve progression in our previous study,^16^ and the current study further indicated the importance of targeting asymmetrical expression of ESR1 to treat AIS from a clinical perspective. Thus, targeting the decreased ESR1 expression in concave paraspinal muscle progenitor cells for AIS could be a promising research field with clinical translational significance in the future study.

The current study was not without limitations. Firstly, this was a single‐center study and the sample size was not large. A multi‐center study with larger AIS patients was required to confirm the validity of the conclusion of the current study. Secondly, all paraspinal muscle tissues were obtained from patients with the Cobb angle larger than 40 degrees. If muscles from patients with less severe scoliotic deformity could be obtained after ethical approval, more information could be revealed to draw a conclusion. Furthermore, this was a cross‐sectional design study, which restricted the ability to establish a causal relationship between muscle ESR1 and AIS. However, since humans are the exclusive species to develop AIS, there are no alternative models that accurately emulate its developmental mechanisms.

## 5 CONCLUSION

There were 48% of AIS patients with significantly decreased ESR1 expression of concave paraspinal muscle progenitor cells (convex/concave >1.5 folds), while patients with more asymmetrical ESR1 expression showed more hypoplastic paraspinal muscle and fatty infiltration on the concave side, and more severe scoliotic deformity.

## CONFLICT OF INTEREST STATEMENT

The authors declare no conflicts of interest.
